# The First Outbreak of Novel Coronavirus Disease 2019 (COVID-19) at an Outdoor Camping Site in South Korea, 2020

**DOI:** 10.2188/jea.JE20230051

**Published:** 2024-04-05

**Authors:** Na-Young Kim, Seonhee Ahn, GwangJin Kim, Donghyok Kwon, Young-Joon Park, Sang-Eun Lee

**Affiliations:** 1 Central Disease Control Headquarters, Korea Centers for Disease Control and Prevention Agency (KDCA), Cheongju, Republic of Korea; 2 Homicide, Gwangju Nambu Police Station, Gwangju, Republic of Korea

Novel coronavirus disease 2019 (COVID-19) is transmitted through the respiratory tract of infected individuals^1^^,^^2^ and air transmission in an enclosed space.^3^ In South Korea, many COVID-19 outbreaks occurred in closed spaces, crowded places, and close-contact environments (3Cs environment),^4^^,^^5^ such as workplaces,^6^ fitness centers,^7^^–^^9^ and nursing homes.^10^ However, an outbreak occurred at an outdoor camping site in 2020, raising the need to observe precautions even when outdoors. We aimed to investigate the epidemiological characteristics and transmission risk factors for developing precautionary measures during outdoor activities.

This study analyzed the COVID-19 outbreak among families who camped together from July 24–26, 2020. The epidemiological characteristics were obtained through an investigation; the transmission risk was evaluated through additional site visits and closed-circuit television (CCTV) analysis. Data of confirmed cases were collected and placed in Microsoft Excel 2020 (Microsoft Corp., Redmond, WA, USA).

Eighteen people (6 families) participated, with each family comprising three members (parents and a child); 10 people (55.6%) were confirmed to have COVID-19 among them. The index case developed fever on July 28. Among the confirmed cases, eight patients developed symptoms, such as fever, cough, sore throat, sputum, and muscle pain. Two cases were asymptomatic at the time of diagnosis. The campground was largely divided into two sections and was completely separated (Figure 1). Section 1 was a yard covered with crushed stone and Section 2 was a wooden floor. Public play facilities included a swimming pool and trampoline. Other public facilities included a sink and a male/female toilet and shower for each area. Each family moved to the campsite using their own vehicles, and installed three shade curtains and six tents in Section 1. The companions of Section 1 were involved in close, repeated conversations, eating, and drinking with the index case during the camping period, without properly following the prevention rules, such as incorrect wearing of masks while staying at outdoor tables and tents. The other 18 participants (6 teams) who used Section 2 of the same campsite during the same period were placed under passive surveillance after confirming that they had no contact with those who stayed in Section 1 based on the CCTV for the 3 days of the camping period and the statements obtained from the users. No additional confirmed patients were identified.

**Figure 1.**
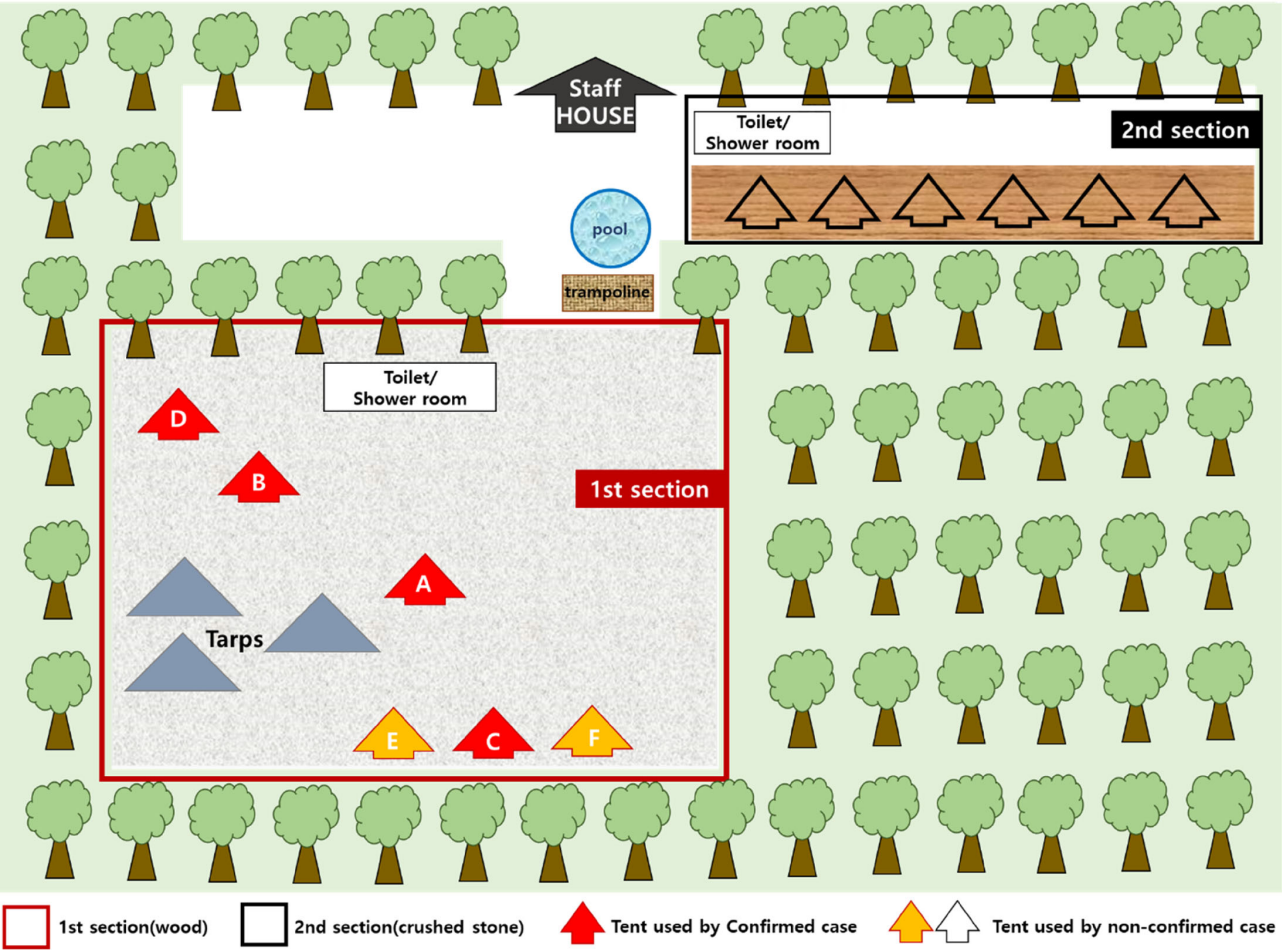
Map of campground and distribution of tents used by confirmed cases

This campsite case is the first outdoor COVID-19 outbreak that occurred in South Korea, with similarities to the overnight camp of Georgia^11^ and the barbecue party of Japan.^12^ Transmission to other participants possibly occurred during the camping period through the routes listed above. The evidence supporting the transmission is as follows: first, the only common place of exposure was identified as the campsite based on the location tracking by global positioning system, the history of visiting hospitals, credit card usage information for the confirmed cases without contact with previously confirmed cases; second, the region did not have an outbreak prior to the occurrence of the group; third, none of the other campers who did not have any contact with the cases were infected while staying in the same campsite at the same time.

As the COVID-19 pandemic period is prolonged, camping is being sought out as a popular outdoor activity that can comply with social distancing without the concern for the spread of an infectious disease.^13^^,^^14^ This outbreak showed that COVID-19 can spread even when outdoors: prevention and control measures, such as maintaining social distancing and wearing a mask, should be observed when a large number of people gather outdoors. COVID-19 variants are continuously emerging, and Omicron is more transmissible than previously circulating variants. To ensure safe camping, facility managers should maintain cleaning and hygiene protocols, and users should minimize the risk of infection by following the recommended precautions while staying in public spaces.

## References

[1] Centers for Disease Control and Prevention (CDC). COVID-19: How COVID-19 Spreads. https://www.cdc.gov/coronavirus/2019-ncov/prevent-getting-sick/how-covid-spreads.html; 2020 Accessed 14.07.2021.

[2] Centers for Disease Control and Prevention (CDC). Clinical questions about COVID-19: questions and answers. https://stacks.cdc.gov/view/cdc/89817; 2020 Accessed 08.06.2022.

[3] Noorimotlagh Z, Jaafarzadeh N, Martínez SS, . A systematic review of possible airborne transmission of the COVID-19 virus (SARS-CoV-2) in the indoor air environment. Environ Res. 2021;193:110612. 10.1016/j.envres.2020.110612 33309820 PMC7726526

[4] Ministry of Health, Labour and Welfare. Information on health and medical consultation. https://www.mhlw.go.jp/stf/covid-19/kenkou-iryousoudan_00006.html; 2020 Accessed 04.04.2022.

[5] World Health Organization. Considerations for implementing and adjusting public health and social measures in the context of COVID-19. https://www.who.int/publications/i/item/considerations-in-adjusting-public-health-and-social-measures-in-the-context-of-covid-19-interim-guidance; 2021 Accessed 04.04.2022.

[6] Park SY, Kim YM, Yi S, . Coronavirus disease outbreak in call center, South Korea. Emerg Infect Dis. 2020;26:1666–1670. 10.3201/eid2608.201274 32324530 PMC7392450

[7] Bae S, Kim H, Jung TY, . Epidemiological characteristics of COVID-19 outbreak at fitness centers in Cheonan, Korea. J Korean Med Sci. 2020;35(31):e288. 10.3346/jkms.2020.35.e288 32776726 PMC7416003

[8] Jang S, Han SH, Rhee JY. Cluster of coronavirus disease associated with fitness dance classes, South Korea. Emerg Infect Dis. 2020;26:1917–1920. 10.3201/eid2608.200633 32412896 PMC7392463

[9] Shin SH, Park E, Kim S, . COVID-19 outbreak and risk factors for infection in a taekwondo gym in the Republic of Korea. Osong Public Health Res Perspect. 2022;13:162–170. 10.24171/j.phrp.2021.0295 35538688 PMC9091639

[10] Choi G, Kim NY, Lee SY, . An experience of the early stage of COVID-19 outbreak in nursing homes in Gyeonggi Province, Korea. Korean J Clin Geri. 2022;23:27–35. 10.15656/kjcg.2022.23.1.27

[11] Szablewski CM, Chang KT, Brown MM, . SARS-CoV-2 transmission and infection among attendees of an overnight camp—Georgia, June 2020. MMWR Morb Mortal Wkly Rep. 2020;69:1023–1025. 10.15585/mmwr.mm6931e1 32759921 PMC7454898

[12] National Institute of Infectious Diseases: Field Epidemiology Training Program (FETP). Outbreak of COVID-19 in “Leisure Activity such as Hobbies”. Center for Surveillance, Immunization, and Epidemiologic Research (CSIER), National Institute of Infectious Diseases Japan. https://www.niid.go.jp/niid/ja/diseases/ka/corona-virus/2019-ncov/2484-idsc/10005-covid19-27.html; 2020 Accessed 22.03.2023 (in Japanese).

[13] Kim JH, Lee HR. Comparative study on the changes in perceptions of camping in tourists before and after the COVID-19 outbreak using social media big data: focused on semantic network analysis. Korean J Hospitality Tourism. 2021;30:117–134 (in Korean). 10.24992/KJHT.2021.7.30.05.117

[14] Rice WL, Mateer T, Taff BD, . The COVID-19 pandemic continues to change the way people recreate outdoors: a second preliminary report on a national survey of outdoor enthusiasts amid the COVID-19 pandemic. SocArXiv. 2020;1:1–15.

